# From Food to Genes: Transcriptional Regulation of Metabolism by Lipids and Carbohydrates

**DOI:** 10.3390/nu13051513

**Published:** 2021-04-30

**Authors:** Inés Bravo-Ruiz, Miguel Ángel Medina, Beatriz Martínez-Poveda

**Affiliations:** 1 Andalucía Tech, Departamento de Biología Molecular y Bioquímica, Facultad de Ciencias, Universidad de Málaga, E-29071 Málaga, Spain; inesbravoruiz@hotmail.com (I.B.-R.); medina@uma.es (M.Á.M.); 2 Instituto de Investigación Biomédica de Málaga (IBIMA), E-29071 Málaga, Spain; 3 CIBER de Enfermedades Raras (CIBERER), E-29071 Málaga, Spain; 4 CIBER de Enfermedades Cardiovasculares (CIBERCV), E-28029 Madrid, Spain

**Keywords:** ChREBP, lipid and glucose metabolism, macronutrient sensing, Mondo, nutrigenomics, PPAR, transcriptional regulation

## Abstract

Lipids and carbohydrates regulate gene expression by means of molecules that sense these macronutrients and act as transcription factors. The peroxisome proliferator-activated receptor (PPAR), activated by some fatty acids or their derivatives, and the carbohydrate response element binding protein (ChREBP), activated by glucose-derived metabolites, play a key role in metabolic homeostasis, especially in glucose and lipid metabolism. Furthermore, the action of both factors in obesity, diabetes and fatty liver, as well as the pharmacological development in the treatment of these pathologies are indeed of high relevance. In this review we present an overview of the discovery, mechanism of activation and metabolic functions of these nutrient-dependent transcription factors in different tissues contexts, from the nutritional genomics perspective. The possibility of targeting these factors in pharmacological approaches is also discussed. Lipid and carbohydrate-dependent transcription factors are key players in the complex metabolic homeostasis, but these factors also drive an adaptive response to non-physiological situations, such as overeating. Possibly the decisive role of ChREBP and PPAR in metabolic regulation points to them as ideal therapeutic targets, but their pleiotropic functions in different tissues makes it difficult to “hit the mark”.

## 1. Introduction

The discovery of the lactose operon in bacteria first explained the relationship between enzymatic activity and the transcriptional control of gene expression [1]. Today, transcriptional regulation is known as an essential control system for all organisms. Evolution has favored the development of different mechanisms to efficiently store nutrients under circumstances of plenty food availability, making them available for use in periods of shortage. In the case of glucose, strict control allows the maintenance of constant plasma levels that guarantee its primordial energy contribution for many cells such as erythrocytes or organs such as the brain. Unfortunately, this harmonious network of regulatory systems that govern metabolic homeostasis is disrupted in obesity and related diseases such as type 2 diabetes mellitus (T2DM), fatty liver disease or metabolic syndrome. These diseases are serious epidemics of the 21st century, due to their high prevalence in the population and their socioeconomic damage.

As life expectancy increases, so does the emergence of these diseases, typical of Western societies and largely a consequence of today’s lifestyle. Major advances in science, for example, with the high performance of omics techniques or the applicability of the interrelationship of systems biology, are increasingly facilitating the study of all diseases and drug development.

In this context, it is very interesting to study the nutritional regulation of gene expression by pointing to the foods we eat, the implicated genes and the metabolic pathways as the vertices of a “Bermuda triangle” that still hides some mysteries of metabolic regulation and its pathological deregulation. From this paradigm, nutritional genomics arises in its two faces of nutrigenomics (aiming to explain the direct effect of nutrients on gene expression) and nutrigenetics (aiming to understand how genetic variants predispose the metabolism of ingested nutrients). Nutrigenomics field especially has experienced a great advance in the recent years regarding the beneficial epigenetic effects of some micronutrients and phytochemical components of the diet [2], leading to their pharmacological use as “nutraceuticals” [3]. On the other hand, macronutrients’ role in gene transcription may not seem as attractive as the epigenetic regulation by phytochemicals since they are at the core of complex metabolic networks. Nonetheless, we consider that glucose and lipids, as major components of our diet, should not be overlooked and what is more, that deciphering their nutrigenomic actions in both physiological and pathological conditions would greatly contribute to human health.

Hence, the aim of the present article is to review the state of knowledge of gene regulation by lipids and carbohydrates, with a special focus on peroxisome proliferator-activated receptors (PPARs) and carbohydrate response element binding protein (ChREBP) pathways. Specifically, the mechanisms of action of these factors and the metabolic functions in which they participate are studied in depth, considering their possible use for pharmacological intervention.

## 2. Gene Expression Regulation by Lipids

The role of lipids in the organism has been the subject of ongoing research. As the regulatory mechanisms of lipid metabolism were detailed, novel functions of unsaturated fatty acids and their derivatives were discovered; for example, in the cognitive development [4] or in the immune response [5]. It has been observed that an unbalanced intake of saturated and unsaturated fat, or even an imbalance in the type of polyunsaturated fat [6], is commonly associated with many different diseases, including those with cardiovascular etiology [7] or metabolic origin such as diabetes [8] or even many types of cancer [9]. Initially, it was thought that fatty acids modulated such processes solely on the basis of their structural function, with the modification of the lipid composition of the membrane affecting signal transduction, and their energetic function. However, at the end of the 20th century, a new actor joined the scene: the gene regulation directly induced by fatty acids. This finding was deduced from evidence suggesting that fatty acids could function similarly to hydrophobic hormones, binding to and activating nuclear receptors. The nuclear receptors, in turn, would act as transcription factors modulating the expression of numerous genes [10].

### 2.1. Fatty Acid Sensors: Nuclear and Membrane Receptors, and Transcription Factors

The major lipid response factors belong to the nuclear receptor family, including PPARs, liver X receptors (LXR), hepatocyte nuclear factor 4 alpha (HNF4α), and retinoid X receptor (RXR).

**Figure 1:**
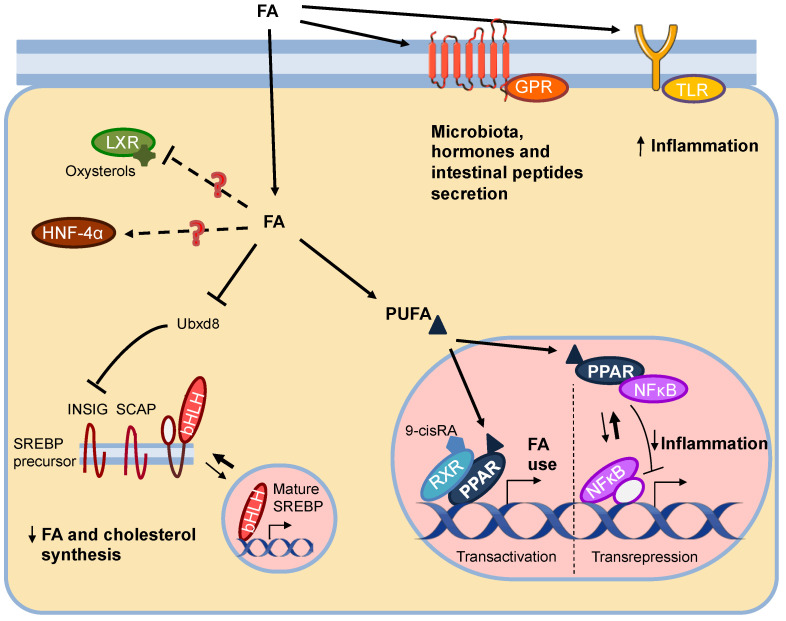
General mechanisms of transcriptional regulation by fatty acids (FA). Fatty acids bind to the TLR4 or GPR membrane receptors, inducing inflammation or hormone secretion, respectively. They can also bind to nuclear receptors, although their interaction with LXR and HNF-4α is not entirely clear. In contrast, PUFA binding to PPAR can induce transactivation from the formation of the active heterodimer PPAR-RXR, which promotes different pathways of fatty acid use, or transrepression, recruiting NF-κB and preventing its action on its target genes and thus reducing inflammation. Regarding transcription factor SREBP, its maturation is repressed by fatty acids through inhibition of Ubxd8 and promoting the sequestration of SREBP by SCAP and INSIG, among other mechanisms. This figure was created with Servier Medical Art (https://smart.servier.com/, accessed on 20 August 2020) under a creative commons license (https://creativecommons.org/licenses/by/3.0/).

The family of nuclear receptors is structurally very well conserved. Its structure is based on six regions (A/B, C, D, E, F) including the activation factor domains 1 and 2 (AF-1, AF-2) where coregulatory proteins bind, the DNA-binding domain (DBD) by which the receptor binds to its response element in the target genes and the ligand-binding domain (LBD) [11]. The first described example of a lipid-regulated nuclear receptor is PPAR. Three types of this receptor are known, PPARα, PPARβ, and PPARγ. All of them are activated by fatty acids, preferentially by long-chain polyunsaturated fatty acids (PUFA), as well as by some fatty acid derivatives such as eicosanoids and acyl coenzyme A (acyl-CoAs) [12] (Figure 1). Each type of PPAR has tissue-dependent expression and function that are complementary in the regulation of lipid metabolism in order to ensure homeostasis [13]. PPARs are the best described examples of lipid nutrigenomics and therefore will be discussed in detail in Section 2.2.

Regarding LXRs, there are two subtypes: LXRα with a dominant expression in liver, adipose tissue and macrophages, and LXRβ more widely expressed among all tissues [14]. These receptors carry out essential functions in lipid anabolism: they promote the synthesis of bile acid and cholesterol, lipogenesis, reverse cholesterol transport, and fatty acids and glucose uptake. Their endogenous ligands are oxysterols, such as 24(S)-hydroxycholesterol and 24(S),25-epoxycholesterol [15]. Moreover, some studies have raised the possibility that unsaturated fatty acids could compete with oxysterols for LXR and antagonize their function [16] (Figure 1). This could be an explanation for the existing connection between PUFAs and the reduction of an important LXR target gene, the sterol regulatory element binding protein (SREBP), although it is not the only mechanism described [12]. Therefore, the role of LXR as a direct sensor of fatty acids is less evident than in the case of PPAR, although it is not excluded that LXR can be otherwise regulated by fatty acids. In fact, the presence of elements of response to PPAR has been described in the promoter region of LXRα gene [17].

The case of HNF-4α regulation directly by fatty acids is even more controversial than that of LXR (Figure 1). This nuclear receptor regulates the expression of several apolipoproteins, glycolytic enzymes, and CP450 monooxygenases, among others [18]. Initially, several studies proposed that fatty acyl-CoAs were ligands of this receptor and exerted opposite effects on gene expression depending on whether they were saturated or unsaturated fatty acids [19]. However, later, X-ray crystallography studies showed important differences in the structure and functioning of HNF-4α compared to others regulated by fatty acids, such as a much smaller pocket size or the presence of fatty acids constitutively bound to the LBD of the receptor, suggesting its function as an integral part of the receptor [20]. Although some fatty acids like linoleic acid have been described as endogenous ligand to HNF-4α [21] no strong evidence supports that fatty acids or derivatives could modulate gene transcription from HNF-4α.

The RXR nuclear receptor is best known for its function as a coreceptor. This promiscuous receptor participates in most of the heterodimers to conform the active complex that bind to response elements in the DNA, including PPAR-RXR (Figure 1). Although the main RXR ligand is 9-cis-retinoic acid, it is important to note that RXR exhibits certain affinity for docosahexaenoic acid (DHA), a PPAR ligand [22], meaning that DHA could bind to either of the two receptors of the PPAR-RXR heterodimer.

In addition to the nuclear receptors, there are other transcription factors that could also be directly regulated by lipids. The main factor described is SREBP, which includes SREBP-1a, SREBP-1c and SREBP-2, with SREBP-1 preferably involved in de novo lipogenesis while SREBP-2 controls cholesterol synthesis [23]. SREBP is translated as a precursor in the endoplasmic reticulum membrane, where it is retained by the SREBP cleavage activator protein (SCAP) and insulin-induced proteins (INSIG). When the level of sterols decreases, INSIG proteins are ubiquitinated and degraded via proteasome and the SREBP-SCAP complex is transported to the Golgi apparatus where some proteases perform the cut of the precursor form allowing the translocation of the mature SREBP to the nucleus [24]. In the nucleus, this transcription factor promotes the synthesis of fatty acids and cholesterol [16]. There is evidence that PUFAs can interact with SREBP-1c and inhibit its activity. The proposed mechanisms of this inhibition occur at different levels [12]. On the one hand, PUFAs inhibit the ubiquitin regulatory X domain-containing protein 8 (Ubxd8) that mediates the degradation of Insig-1 and therefore, stabilizes Insig-1 preventing the maturation of SREBP [25] (Figure 1). On the other hand, DHA seems to favor the degradation of SREBP by the proteasome [26]. In addition, a correlation between PUFAs and a decreased transcriptional activity of SREBP is observed. This could occur either by self-regulation of SREBP in response to the repression of its maturation [27] or by the already mentioned antagonism of PUFAs to LXR, that activates SREBP gene transcription [28].

Another fatty acid-regulated transcription factor is carbohydrate response element binding protein (ChREBP), which will be analyzed in depth in Section 3.

Finally, other important proteins involved in lipid recognition, such as Toll receptor type 4 (TLR4) or certain G-protein associated receptors (GPRs), should be mentioned (Figure 1). Unlike the factors explained above, these receptors bind their ligands in the extracellular space, eliciting signaling cascades from cell surface which finally modulate gene expression. TLR4 is an extracellular saturated fatty acid receptor that modulates inflammatory processes, also contributing to insulin resistance [29]. With respect to GPRs, some appear to mediate metabolic functions related to microbiota, such as GPR41 and GPR43, which respond to short chain saturated fatty acids and are located in the colon [30]. Other receptors such as GPR40, and GPR120, which are activated by long unsaturated fatty acids, stimulate insulin secretion in pancreatic cells [31] or other gastrointestinal hormones in enteroendocrine cells [32], respectively.

### 2.2. PPARs

#### 2.2.1. PPARs and Their Ligands

The history of these nuclear receptors began with the discovery that certain pesticide molecules and fibrates, previously used for their hypolipidemic effect, produced the proliferation of peroxisomes in mice, leading to hepatomegaly and cancer [33]. In 1990, the molecule responsible for these effects was identified as a new member of the steroid hormone superfamily with considerable differences with other members described and was named PPAR because of its observed effects [34]. Years later, it was renamed PPARα, due to the discovery of their structural homologues PPARβ and PPARγ in *Xenopus laevis* [10]. Later, thiazolidinedione drugs were associated with an agonist effect on PPARγ, which is the molecular reason for its therapeutic action against T2DM [35]. Although some of these drugs were discarded due to toxicity, pioglitazone and rosiglitazone have been used until today [36]. Currently, new actions of these nuclear receptors continue to be discovered in various diseases while investigating dietary ligands in nutrigenomics field.

PPARs’ natural ligands are fatty acids and their derivatives, which can come from diet, de novo lipogenesis, or complex lipolysis [37]. Unsaturated fatty acids are ligands of the three PPARs, although there is preference for PUFAs due to structural factors [38] and availability within the intracellular pool of fatty acids [39,40] (Table 1). Regarding their oxidized derivatives as PPARs ligands, eicosanoids predominate, obtained from 22-C PUFAs by the activity of cyclooxygenases (COX) and lipoxygenases (LOX). Some important ligands of this group are hydroxy eicosatetraenoic acid (HETE), specially 8- and 15-HETE and leukotriene B4 (LTB4) [41,42,43]. In addition, oxidized derivatives of linoleic acid activate PPARs, notably 9-hydroxyoctadecadienic acid (9-HODE) and 13-HODE [37,44]. Among these derivatives, some are specific to a certain PPAR: LTB4 to PPARα [45], 15-desoxi-delta-12,14-prostaglandin J2 to PPARγ [46] and prostacyclin I2 to PPARβ [47] (Table 1). In addition, new fatty acids or other molecules relatively similar in structure, such as terpenes, polyphenols, and alkaloids, are continually being described to activate these receptors, indicating that PPARs can recognize a wide number of molecules [48]. This promiscuity is due to the size of the LBD pocket of these receptors and is a distinguishing feature against other very specific nuclear receptors [37]. This fact is of great interest since, instead of assuming a non-optimized functioning by non-specific activation, it could facilitate the integral response of the lipid metabolism, as is explained below.

**Table 1:** Nutrient-derived and synthetic activating agents of PPARs and ChREBP.

Receptor.	Nutrient-Derived Activating Agents	Synthetic Activating Agents
PPARα	Saturated and unsaturated fatty acids [38,41] Leukotriene B4 [45] 8-hydroxyeicosatetraenoic acid [41,42]	Fibrates [33]
PPARβ	Polyunsaturated fatty acids [38,41] 15-hydroxy-eicosatetraenoic acid [43] Prostacyclin [47]	GW501516
PPARγ	Polyunsaturated fatty acids [38] 15-deoxy Δ12,14-prostaglandin J2 [46] 15-hydroxyeicosatetraenoic acid [37,44] 9- and 13-hydroxyoctadecadienoic acid [37,44]	Thiazolidinediones [35]
MondoA, ChREBPα/β	Glucose-6-phosphate [49] Xylulose-5-phosphate [50] Fructose-2,6-bisphosphate [51]	------

#### 2.2.2. PPAR Mechanism of Action

PPARs play their role as transcription factors in the form of a heterodimer with RXR [52]. The heterodimer binds to the PPAR response element (PPRE) in promoter regions of the genome, consisting in repeats of the “AGGTCA” motif separated by a nucleotide, and because of its spatial configuration is called DR1. The three PPARs bind to the canonical DR1 in the different target genes of the cell types where each is expressed. However, since in some cells there may be a coexpression of two types of PPAR, the question arises as to how specific recognition of the target gene by each factor takes place. In any case, the fact is that most PPAR response genes can be activated by all the three subtypes, although the 5′ flanking region of RD1 may indicate some specificity [53].

The activation of the PPAR nuclear receptors by their ligands is caused by conformational changes, specifically a transposition of the AF2 domain helix 12, allowing the dissociation of corepressor factors and the association of coactivators [11] promoting the transcription of target genes. In addition, it has been reported that the heterodimer PPAR-RXR acquires active conformation with the binding of the RXR ligand (9-cis retinoic acid or fatty acids), in absence of lipid binding to PPAR, although this activation would be weaker than the elicited by PPAR-ligand binding [54].

Regarding the possibilities of gene expression regulation, PPARs follow the canonical model explained above, which is called “transactivation” (Figure 1). In some cases, the binding of the nuclear receptor may favor repression of the gene rather than its transcription. However, the mechanisms of negative regulation by PPARs are usually indirect. Indirect mechanisms are those where the action of PPARs is not a result of target gene binding, but rather PPARs interact with other factors that directly regulate gene transcription. This is known as “transrepression” and mediates most of the anti-inflammatory actions of PPARs [37], as it is explained in Section 2.2.4.

#### 2.2.3. PPAR Metabolic Functions

##### PPARα

**Figure 2:**
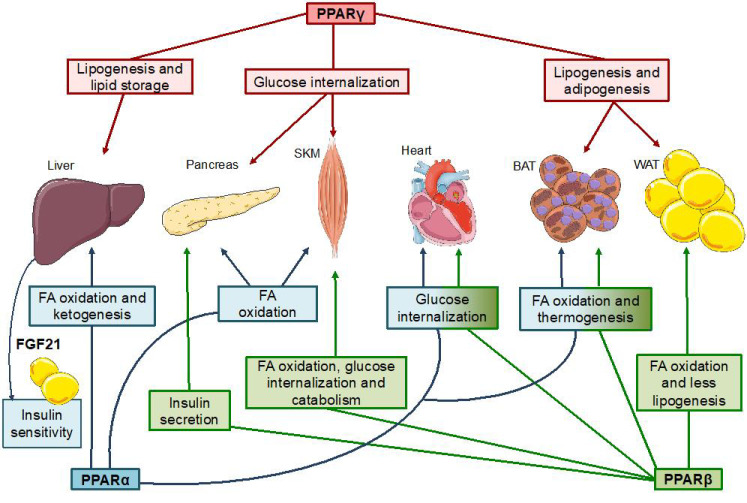
Metabolic functions of PPAR in the main organs and tissues. The three PPARs coordinate glucose and fatty acid (FA) homeostasis by acting on the liver, pancreas, skeletal muscle (SKM), heart, brown adipose tissue (BAT) and white adipose tissue (WAT), essentially. The actions of PPARγ are shown in red, PPARα in blue and PPARβ in green. This figure was created with Servier Medical Art (https://smart.servier.com/, accessed on 20 August 2020) under a creative commons license (https://creativecommons.org/licenses/by/3.0/).

This receptor is essential for the oxidation of fatty acids and is expressed in oxidative tissues, mainly in the liver, where its function is better characterized, and secondarily in heart, kidney and brown adipose tissue [55]. Its main role described is the adaptation to the post-absorptive state, while under nutrient availability conditions, detected by the target complex 1 of rapamycin in mammals (mTORC1), it remains inhibited [56]. In order to maintain energy homeostasis during fasting, PPARα induces the expression of multiple enzymes related to the entry of fatty acids to the mitochondria and β-oxidation (lipoprotein lipase, carnitine palmitoyltransferase, acyl-coA oxidases and dehydrogenases), and the generation of glucose or ketone bodies (hydroxymethyl glutaryl CoA synthase) in the liver [57] (Figure 2). Besides, PPARα induces the secretion of the hepatokin fibroblast growth factor 21 (FGF21) which has been described to act in adipose tissue improving insulin sensitivity and ameliorating whole body glucose metabolism [58]. It also promotes fatty acid oxidation in other organs and thermogenesis in brown adipose tissue [59] (Figure 2). In addition, this receptor regulates the expression of apolipoproteins by favoring the decrease of low-density (LDL) and very low-density lipoproteins (VLDL) and the increase of high-density lipoproteins (HDL) [60]. Therefore, fibrates (agonists of this receptor) are used to decrease triglycerides in blood, which would as a result improve insulin sensitivity [61]. The opposite effect occurs in PPARα knocked-out mice, which exhibit a phenotype that includes hypoglycemia, hypoketonemia and hypertriglyceridemia [62]. Interestingly, some studies show that PPARα is only activated by fatty acids newly incorporated from the diet or synthesized de novo (known as “recent fat”) but not by fatty acids from adipocyte triglycerides, called “old fat” [63].

##### PPARγ

This receptor has two subtypes: PPARγ1 and PPARγ2. While PPARγ2 is expressed exclusively in adipose tissue, PPARγ1 is found in other locations, especially in macrophages [64]. In any case, it is a crucial factor in the formation and maintenance of adipose tissue, so that in PPARγ knocked-out mice the differentiation of fibroblasts to adipocytes is prevented [65] (Figure 2). In addition, both the murine knockout specific to adipose tissue [66] and the human negative dominant mutation in PPARγ [67] are associated with lipodystrophy and insulin resistance. The primary functions of PPARγ are therefore activation of lipogenesis and insulin sensitization (Figure 2). On the basis of this second role, thiazolidinediones, agonist drugs of this receptor, were developed for the treatment of T2DM [37]. One of the mechanisms by which this receptor promotes insulin sensitization is the transcription of enzymes and transporters that allow lipogenesis and lipid storage in adipose tissue (CD36, fatty acid transport proteins (FATP), SREBP) resulting in less circulating fatty acids and triglycerides [13]. Interestingly, PPARγ expression in the brain accounts for thiazolidinedione-related insulin sensitivity and weight gain [68]. In addition to the redistribution of lipids, PPARγ also inhibits the synthesis of tumor necrosis factor α (TNFα) [69] and resistin [70], both related to inflammation and insulin resistance, while inducing the synthesis of adiponectin, which favors the oxidation of fatty acids [71]. On the other hand, there is evidence of direct antidiabetic effect of PPARγ in pancreas and skeletal muscle by favoring the uptake of glucose through activation of calbindin (CABP) and GLUT4 transporter [72]. In brown adipose tissue, PGC-1α, the coactivator of PPARγ, promotes mitochondrial biogenesis [73].

##### PPARβ

Its expression is ubiquitous, yet to a lesser extent in the liver. This receptor, although less studied than the other two members of the family, shows a metabolic role opposite to PPARγ and similar to PPARα. PPARβ promotes the oxidation of fatty acids [13], while PPARα is essential for adaptation to the fasting state, this receptor is essential in adaptation to exercise. It is especially relevant in skeletal muscle, where the expression of PPARα is low. In this tissue, PPARβ enables the oxidation of fatty acids in addition to promoting the transformation into type I oxidative muscle fibers and increasing the incorporation of glucose by GLUT4 [74] (Figure 2). Similarly, in the cardiac muscle, oxidative metabolism and mitochondrial synthesis are promoted by PPARβ, improving cardiac function [75]. In the pancreas it also contributes to oxidative metabolism and insulin secretion [76] (Figure 2). On the other hand, it inhibits lipogenesis in the liver through the destabilization of SREBP-1c and favors the oxidation of fatty acids in the adipose tissue [77] (Figure 2). Regarding the brown adipose tissue, it promotes thermogenesis through the uncoupling protein (UCP-1) [78] (Figure 2). Mice with overexpression of this receptor show resistance to obesity and a phenotype of marathon runners, in contrast with the tendency to overweight exhibited by knocked-out mice Hence, PPARβ could have a promising potential for the treatment of obesity, T2DM and cardiovascular disease. However, clinical trials of GW501516, a strong agonist of PPARβ, were suspended after the carcinogenic effect observed in rats [79].

#### 2.2.4. PPAR Anti-Inflammatory Functions

The most studied PPAR-related anti-inflammatory mechanism is the direct interaction with the transcription factor NF-κB preventing its binding to the promoters of its response genes (Figure 1). Nonetheless, PPARs repress NF-κB transcriptional action in many ways, such as inducing its degradation through E3 ubiquitin ligase, increasing the expression of its repressor IκBα, promoting the activity of the deacetylase SIRT1 or in a transrepression fashion by binding p300 and hampering its NF-κB coactivation action [80]. This transrepression function of PPARs has been described for other transcription factors apart from NF-κB. An interesting example is the repression of the inflammatory interleukin-6 (IL-6) gene resulting from PPARα binding to either NF-κB, CBP or c-Jun [81]. In addition, PPARs also induce anti-inflammatory actions by regulating reactive oxygen and nitrogen species (ROS; NOS), sometimes by direct gene transcription regulation or through other indirect mechanisms. Several antioxidant enzymes’ expression is increased in response to PPAR, being a PPRE characterized in the promoter of the enzymes’ genes, as is the case of superoxide dismutase [82] and catalase [83]. On the contrary, PPARs downregulate RNS levels by inhibiting their generation sources. As an example, PPARγ has been shown to repress inflammatory gene expression, as is the case of the gene of inducible nitric oxide synthase (iNOS), by the stabilization of inhibitory protein complexes [84].

Many of these PPARs transrepression-induced anti-inflammatory actions resemble those of glucocorticoids [85]. This translates into the possibility of developing new drugs for the treatment of inflammation-dependent diseases or the dietary-based regulation of the immune system. For example, the palmitoylethanolamide, a natural compound which reduces pain and inflammation, is known to act through PPARα [86]. However, there is evidence that PPARs can mediate proinflammatory actions as well. Apparently contradictory to NF-κB PPARs-induced repression, PPARγ is known to induce the expression of COX-2, since a PPRE is known in *PTGS2* promoter [87]. This is most likely related to the important biological function of prostanoids following the rationale of a substrate, PUFAs, promoting the expression of its processing enzyme, COX-2 [80]. Nonetheless, PPARβ can also upregulate COX-2 expression and PGE2 production, subsequently increasing proinflammatory cytokines secretion, along with AKT signaling resulting in a proinflammatory and prosurvival environment which cancer cells take advantage of [88,89]. On this line, PPARα has been associated with stimulus-primed proinflammatory responses, as activating endothelial cells to produce monocyte chemoattractant protein-1 (MCP-1) upon LDL oxidation or increasing TNF levels in an endotoxemia context [90].

Altogether, the three PPARs prevent fatty acids-driven lipotoxicity either by activating their oxidation or by storing them as triglycerides in the adipose tissue. In this way, fatty acids themselves exert a control on their own metabolism through these receptors. Furthermore, PPARs are associated to anti-inflammatory actions which has drawn attention on its potential therapeutic contribution to many diseases (reviewed in [91]). In the pharmacological context, none of the classical drugs have been entirely successful. The beneficial effects of fibrates are undetermined, thiazolidinediones have numerous side effects such as weight gain, edema or heart failure, among others, and GW501516 increases the risk of cancer. Currently, drugs that activate both PPARα and PPARγ such as gliatazars, or selective modulators of PPARs such as pemafibrate for PPARα, are being developed as T2DM and antiatherosclerotic therapy, respectively, and appear to have better results [36]. On the other hand, the contribution of PPARs to inflammation should be further studied and its two pro- and anti-inflammatory faces must be taken into consideration regarding PPAR agonists use in therapy.

## 3. Gene Expression Regulation by Carbohydrates

Glucose is a virtually universal energetic nutrient and also substrate for synthesis of metabolites by the cellular machinery. Therefore, there are numerous mechanisms that keep plasma glucose levels stable despite variations in intake. Traditionally, it was believed that the activation of both glycolytic and lipogenic pathways in tissues after rising glucose in plasma was essentially due to insulin. However, several studies in the 1990s revealed that, in certain genes related to these pathways, the action of insulin was insufficient, or secondary to other regulatory mechanisms directly modulated by glucose [92]. For example, a study with rat hepatocytes showed that glucose and insulin were necessary for the expression of the L-type pyruvate kinase (L-PK) gene [93]. A similar result was obtained for the fatty acid synthase (FAS) complex gene, where the addition of glucose to rat hepatocytes cultured in the presence of insulin and other factors correlated with an increase in the FAS mRNA level, which did not occur if either glucose or insulin were not present [94]. In this same experiment, the role of insulin on FAS expression was found to be indirect, inducing the expression of the enzyme glucokinase, necessary to transform glucose into glucose-6-phosphate (G6P), which would be the potentially activating metabolite of FAS. At the same time, carbohydrate response sites (ChoRE) were characterized in the promoter of some genes of these pathways, being L-PK the first gene where it was identified [95]. By the end of the 1990s, a consensus sequence of ChoRE in these genes was intuited, to which a transcription factor from the leucine zipper family should be attached, and G6P was described as the candidate signaling molecule [92].

Finally, in 2000, the transcription factors MondoA [96] and MondoB, initially designated as WBSCR14 according to its association with Williams-Beuren neurodevelopmental syndrome [97], were discovered. Afterwards, MondoB was characterized as a carbohydrate sensor and properly renamed as ChoRE-binding protein (ChREBP), given its increased expression in a high carbohydrate diet, its specificity for E-box sequences (see Section 3.1) in the L-PK promoter and its tissue location parallel to that of the target genes [98]. In 2012, a new, smaller isoform of ChREBP generated by alternative splicing, was discovered and defined as ChREBP-β [99]. MondoA and the long and short isoforms of ChREBP are involved in the regulation of glycolysis and lipogenesis pathways, directly induced by the binding of glucose derived metabolites. However, they show differences in tissue expression and activation mechanism related to glucose levels, meaning a complex control network of the homeostasis of this macronutrient, which is impaired in diseases such as T2DM. Since its discovery, the mechanism of activation and the function of these factors have been studied in depth, as explained below.

### 3.1. MondoA/ChREBP Structural Features

The Mondo family belongs to the transcription factor superfamily with basic helix-loop-helix and leucine zipper (bHLH/LZ) domain, which includes factors such as Myc or Mad [96]. The action of these proteins is characterized by requiring the formation of a heterodimer with the protein Max, which enables the activation of transcription by the Myc-Max pair, promoting cell growth, and the repression of transcription in the case of the Mad-Max pair, inhibiting proliferation and favoring differentiation [100]. Analogous to Myc-Max, Mondo forms an active heterodimer with the Max-like X protein (MLX) [101], the reason why it is also known as MLX interacting protein (MLXIP) and ChREBP as MLX interacting protein-like (MLXIPL). However, unlike Myc, MondoA and ChREBP are located in the outer membrane of the mitochondrion, so that transcription activation by this factor is in the first place controlled by its translocation to the nucleus [96].

ChREBP’s target genes contain the element ChoRE in their promoter regions, which is composed of a tandem sequence of E-boxes separated by five nucleotides (5′-CACGTGnnnnnCACGTG-3′). The discovery of this region in the L-PK promoter and the S14 gene derived in the assumption that—the then unknown—ChREBP would belong to the Myc family factors, and that the nucleotides between the E-boxes were crucial [102]. Indeed, ChREBP dimerize with MLX [103], as previously mentioned, and two ChREBP-MLX heterodimers join the two E-boxes to form the active transcriptional complex [104].

**Figure 3:**
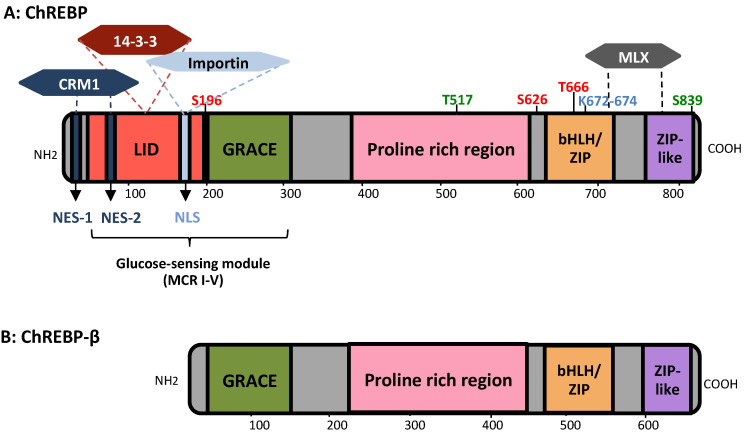
Representation of the structural domains of ChREBP. (**A**) The five ChREBP domains are illustrated in the boxes with an orientation bar of the number of amino acids below. The LID and GRACE domains conform the glucose-sensing module and include the MCR. The NES, NLS, the main target residues of post-translational modifications (phosphorylation in red, acetylation in blue, O-Glc-Acetylation in green) and the binding sites of some of the proteins required for the activation of ChREBP are also indicated. (**B**) The structure of ChREBP-β is analogous to ChREBP except for lacking the first 177 amino acids.

The MondoA and the ChREBP coding genes are located in chromosome regions 12q24.31 and 7q11.23, respectively. The corresponding coded proteins contain 919 and 852 amino acids, respectively, showing a high identity in the C- and N-terminal regions. At the carboxyl end of both proteins, there is a bHLH/LZ domain and a dimerization and cytoplasmic localization (DCD) leucine-like domain that mediates dimerization with MLX [103] and DNA binding. In the central region of the protein there is a sequence rich in proline. The amino end contains two nuclear export signals (NES1, NES2) to which the exportin or chromosomal maintenance protein (CRM1) binds, and a nuclear location signal (NLS) [105]. Between NES2 and NLS, which corresponds to the Mondo conserved region (MCR)III, there is a binding site to α-importin to mediate translocation to the nucleus or, in its absence, to the 14-3-3 protein, responsible for its cytosolic retention [106]. More importantly, this N-terminal end contains the highly conserved glucose recognition module (GSM), consisting of the low glucose inhibitory domain (LID) and a conserved glucose response activation element (GRACE) [107]. The GRACE domain is involved in the transactivation of the Mondo/ChREBP target genes but is highly repressed by LID under low glucose conditions. This repression is explained by a hinge mechanism, where an intramolecular interaction physically prevents the binding to DNA [108] (Figure 3).

In 2012, the ChREBP-β isoform was discovered as a new transcript from an alternative promoter and exon 1b, located upstream of ChREBP exon 1a and connected directly by binding to exon 2. This new isoform is translated from exon 4, generating a protein that lacks the first 177 amino acids and therefore lacking the NES, NLS and LID domains [99] (Figure 3). Although it does not possess the nuclear translocation signals, ChREBP-β exhibits a potent transcriptional activity compared to ChREBP, due to the absence of the LID inhibitory domain. Thus, the ChREBP isoform (or ChREBPα) represses its activity in the presence of low amounts of glucose, while ChREBP-β is constitutively active. On the other hand, Herman et al. proposed that the mechanism of action of ChREBP occurs in two phases, where first ChREBP senses glucose level in plasma and then promotes the expression of ChREBP-β through the ChoRE sequence present in its promoter, inducing a positive feedforward loop of amplification. In turn, recent evidence has emerged suggesting the role of ChREBP-β suppressing the transcription of ChREBP, establishing a negative feedback loop of glucose signaling control [109].

### 3.2. ChREBP Mechanism of Action

**Figure 4:**
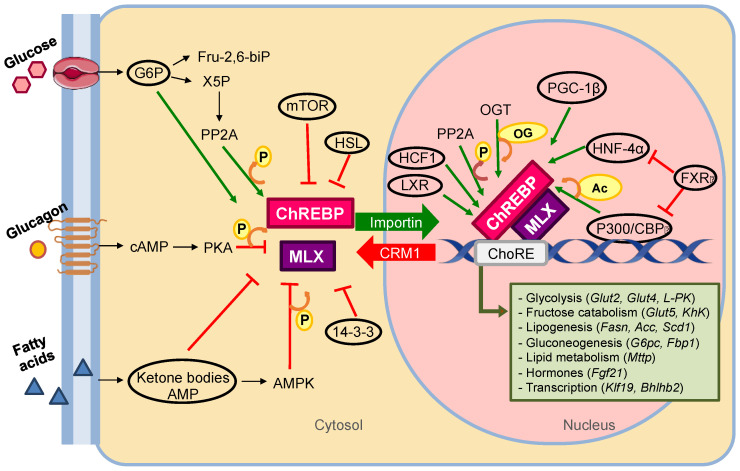
Regulation of nuclear translocation and transcriptional activity of ChREBP. Inactivated ChREBP is located in the cytosol due to phosphorylation by PKA and AMPK and the allosteric inhibition of mTOR, HSL, AMP or ketone bodies. In the presence of glucose, the metabolite G6P induces an active conformation of ChREBP and PP2A, activated by X5P, dephosphorylates it. Partially dephosphorylated ChREBP is translocated to the nucleus, where it undergoes several post-translational modifications important for its activity. The heterodimer ChREBP-MLX binds to several coactivators (such as LXR, HCF1, HNF-4α and p300/CBP, the latter two being repressed by FXR) in order to complete the active transcriptional complex. The proteins included in black circumferences have an allosteric effect, and the yellow circles represent the post-translational modifications of the residues (Ac: acetylation; OG: O-GlcNAcylation; P: phosphorylation). This figure was created with Servier Medical Art (https://smart.servier.com/, accessed on 20 August 2020) under a creative commons license (https://creativecommons.org/licenses/by/3.0/).

The C-terminal region of ChREBP is responsible for the formation of the heterodimer ChREBP-MLX and its binding to DNA, while the N-terminal region contains the glucose sensing element and participates in the cellular localization of the factor. This implies that the action of ChREBP is regulated by two well differentiated mechanisms: nuclear translocation that depends on the binding of importins or exportins, and the induction of transcriptional activity dependent on the formation of the active complex with MLX and the interaction with other cofactors. Many enzymes or factors involved in metabolic regulation mediate the activation (e.g., PP2A, HNF-4α, PGC-1β) or inactivation (e.g., PKA, AMPK, FXR) of ChREBP [110] (Figure 4). However, a distinction should be made between those that have an allosteric effect and those that induce post-translational modifications in ChREBP, such as phosphorylation, acetylation, and O-Glucose-N-acetylation (O-GlcNAcylation) [111]. The main residues where these modifications occur are indicated in Figure 3.

The first regulatory mechanism, phosphorylation, was described at the same time as ChREBP was proposed as the candidate for glucose recognition [98]. In this same study, protein kinase A (PKA) was found to inhibit the transcriptional activity of ChREBP, and protein phosphatase 2 A (PP2A) was shown to restore it. Thus, it was proposed that, under low glucose conditions, the enzyme PKA, induced by glucagon and AMPK (dependent on AMP levels) was activated, being responsible for the repression of ChREBP [112]. PKA phosphorylates ChREBP in Ser196 and Thr666 residues allowing its interaction with 14-3-3 protein and its consequent cytosolic retention. AMPK phosphorylates ChREBP in Ser568 residue, decreasing the binding of this factor to the ChoRE of target genes. Conversely, under conditions of high glucose, the pentose phosphate pathway is promoted and metabolites such as xylulose-5-phosphate (X5P) are increased, activating PP2A. The enzyme PP2A dephosphorylates ChREBP in the Ser196 residue, allowing translocation to the nucleus, where the same phosphatase proceeds to dephosphorylate the Thr666 and Ser568 residues of the factor [50] (Figure 4).

However, this basic model of ChREBP regulation has been questioned through different studies suggesting other metabolites than X5P as signaling agents, and the existence of a phosphorylation-independent mechanism of ChREBP activation [113]. Regarding the metabolites, glucose-6-phosphate (G6P) and fructose-2,6-phosphate (F-2,6-BP) have been proposed as possible ChREBP activators (Table 1). An increase in G6P in β-pancreatic cells INS1 832/13 stimulates the action of ChREBP, and the opposite effect was observed in the case of a decrease in G6P [114]. Therefore, G6P is considered as a ChREBP activating metabolite, although currently there is controversy about its degree of relevance. Some studies propose that cooperation between X5P and G6P is necessary for the activation of ChREBP [115] or that G6P, and not X5P, is the ChREBP signaling metabolite [116]. Likewise, a G6P binding motif is known in the GRACE domain of ChREBP, that would induce an “open” conformation of the factor, where GRACE is released from LID allowing interaction with cofactors and nuclear translocation [49]. In addition to G6P, F-2,6-BP appears to contribute to the activation of ChREBP according to studies showing that the decrease in F-2,6-BP by activation of its phosphatase correlated with the inhibition of ChREBP binding to its target genes [51]. That is, glucose and fructose derived metabolites cooperate in the activation of ChREBP.

On the other hand, the mutation of the phosphorylation sites of ChREBP does not imply the independence of this mutated factor from its activation by glucose, which implies that there must be other underlying mechanisms of action [117]. Acetylation in lysines mediated by histone acetyltransferase [118] and O-GlcNAcylation by adding N-acetylglucosamine to serine/threonine residues [119] (Figure 3 and Figure 4) are involved in the transcriptional activity of ChREBP. Since O-GlcNAcylation depends on substrates produced by the hexosamine biosynthesis pathway, and this in turn depends on glucose and glutamine levels, this modification increases with exposure to high amounts of glucose. This represents an additional glucose-dependent mechanism to regulate the activation of ChREBP, as relevant as O-GlcNAcylation is for ChREBP stability and activity [113].

Apart from post-translational modifications and signaling metabolites, cofactors play a key role in the transcriptional activity of ChREBP. MLX is the main coactivator of ChREBP. However, the allosteric interaction of nuclear receptors with ChREBP is also known [119]. HNF-4α joins the direct repeats of the ChREBP target gene promoter, being an important cofactor for its transcriptional activity [120]. In addition, this ChREBP/HNF-4α complex is stabilized by the coactivator p300 family and cAMP response element-binding (CREB) binding proteins (CBP), which also mediate ChREBP acetylation [121]. On the other hand, FXR functions as a corepressor by binding to the ChREBP/HNF-4α complex inducing the dissociation of ChREBP and p300/CBP histone acetyltransferases and the association of SMRT histone deacetylases to the target gene promoter [122]. Additionally, PGC-1β functions as a coactivator of ChREBP, binding to the target gene promoter and physically interacting with ChREBP [123]. Interestingly, some recent evidence independently points to LXR [124] and HCF-1 [125] as noteworthy coactivators of ChREBP that have shown to enhance its transactivity not only by allosteric interaction, but also by possibly favoring its O-GlcNAcylation (Figure 4). LXR which is O-GlcNAcylated in high glucose context [126] must be unliganded to induce ChREBP coactivation allowing its LBD to bind with LID of ChREBP. Thus, LXR is proposed as a key lipogenesis regulator through LXR response elements when bound to oxysterols or through ChoRE otherwise [124]. For its part, HCF-1 must be O-GlcNAcylated itself as a prerequisite for ChREBP binding further establishing O-GlcNAcylation as a crucial bridge between ChREBP and at least some of the cofactors [125].

Finally, ChREBP signaling is in tune with the metabolic situation through the interaction of ChREBP with different metabolites, receptors and central hormones of metabolism. A study showed that ketone bodies, products of fatty acid oxidation, such as β-hydroxybutyrate and acetoacetate, favor the binding of ChREBP to 14-3-3 protein, preventing translocation to the nucleus [127]. It has also been observed that AMP, whose intracellular level increases during fasting, has a favorable allosteric effect on the interaction of ChREBP and 14-3-3 [128] (Figure 4). This represents a mechanism additional to phosphorylation that ensures inhibition of ChREBP activation under ketosis or fasting situations. On the other hand, insulin seems to indirectly enhance the transcriptional activity of ChREBP, through repression of the Forkhead box protein O1 (FOXO1), which would inhibit O-GlcNAcylation in ChREBP crucial for its stability [129]. The mammalian target of rapamycin (mTOR) kinase [130] and the hormone-sensitive lipase (HSL) enzyme [131] establish allosteric interactions with ChREBP preventing its nuclear translocation. Likewise, interrelationships of ChREBP with several nuclear receptors are known, being LXR [132] and HNF-4α [133] transcription factors that activate the expression of the gene that codes for ChREBP, in addition to their previously mentioned coactivation role.

### 3.3. MondoA/ChREBP Metabolic Functions

**Figure 5:**
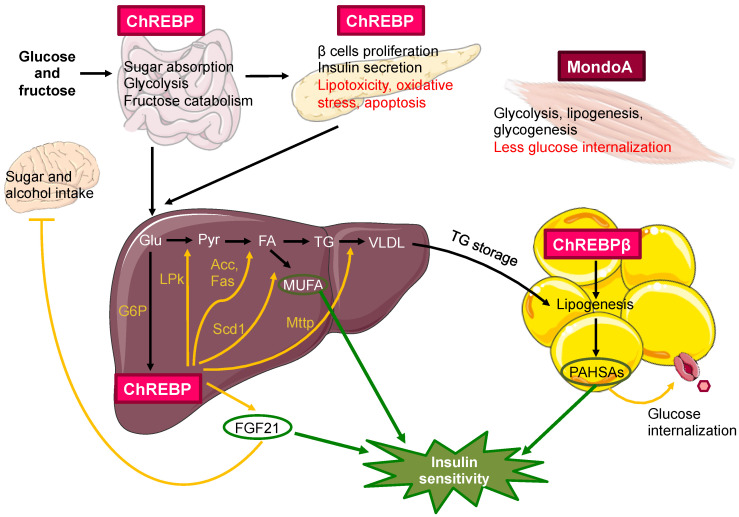
Metabolic functions of ChREBP/MondoA in the main organs and tissues. ChREBP is abundantly expressed in liver and adipose tissue, where it induces glycolysis and lipogenesis represented in the liver. It is also expressed in the intestine and pancreas, where its actions are indicated, highlighting in red the pathological ones. In the skeletal muscle, MondoA stands out. ChREBP promotes the expression of the metabolites MUFA, PAHSA and the hepatokine FGF21, and these are proposed to be responsible for the insulin sensitivity effect. Genes activated by ChREBP are shown in yellow. Pyr: pyruvate; TG: triglycerides. This figure was modified from [111], and created with Servier Medical Art (https://smart.servier.com/, accessed on 20 August 2020) under a creative commons license (https://creativecommons.org/licenses/by/3.0/).

The Mondo family is mainly involved in modulation of genes implicated in glycolysis and lipogenesis, with important target genes such as L-PK, FAS, acetyl-CoA carboxylase (ACC) and steroyl-CoA desaturase (SCD1) [134]. However, while MondoA is essentially located in skeletal muscle regulating glucose metabolism [96], ChREBP is abundant in those tissues that perform lipogenesis such as the liver and adipose tissue, although it is also expressed notably in the pancreas, kidney, skeletal muscle and small intestine [134]. The relevance of ChREBP in the maintenance of health and its link to metabolic diseases has been, and continues to be, a growing issue. In general, ChREBP promotes lipid synthesis, which can contribute to obesity or liver steatosis. However, ChREBP plays an important role in insulin sensitivity by shifting excess glucose into fatty acid production and modulating lipid composition [135]. Therefore, ChREBP is essential in physiological adaptation to overeating and, although an imbalance in the expression of its isoforms may contribute to a pathological state, its pharmacological modulation seems to have more negative consequences than benefits [136]. This is discussed below, highlighting its role in major tissues (Figure 5).

MondoA predominates in skeletal muscle, inducing glycolysis, glycogenesis and lipogenesis in response to increased circulating levels of glucose, while suppressing the uptake of this metabolite by interfering with the insulin signaling. Therefore, MondoA functions as a gatekeeper of glucose homeostasis in muscle but does not contribute to its plasma regulation. High glucose intake increases lipid accumulation in the muscle and insulin resistance, whereas the opposite is observed in mice with muscle-specific MondoA deficiency [137]. Furthermore, SBI-477, an inhibitory molecule of this factor also reduces intramuscular fat and improves insulin signaling, suggesting that MondoA could be a potential therapeutic target in T2DM [138].

In the liver, ChREBP is fundamental since it acts in coordination with SREBP-1c (activated by insulin) in the control of glucose and lipid metabolism. ChREBP knocked-out mice with native activity of SREBP-1c show a normal lipogenic but poor glycolytic enzyme expression. Conversely, SREBP-1c knocked-out mice with normal activity of ChREBP show the opposite phenotype, with normal glycolytic and poor lipogenic enzyme expression [139]. Thus, maximum fatty acid synthesis occurs when insulin and carbohydrates are present. However, in the context of health and disease, the expression of ChREBP in the liver plays antagonistic roles. On the one hand, in obese and insulin-resistant mice, ChREBP seems to be involved in lipogenesis and fatty liver disease, since the knockout of ChREBP in these mice reverses liver steatosis [140]. On the other hand, ChREBP seems to induce the microsomal triglyceride transfer protein (MTTP), essential in the formation of very low-density lipoproteins (VLDL), which favor fat transport from the liver to other tissues [141]. In addition, ChREBP knocked-out mice have worse sensitivity to insulin, so ChREBP is able to dissociate conditions such as hepatic steatosis from insulin resistance and therefore contributes to the maintenance of insulin sensitivity with the counterpart of inducing a fatty liver [135]. It is worth mentioning that this pathological condition of steatosis occurs during the metabolic alteration derived from overfeeding and is probably caused by the activation of the ChREBP-β isoform, which is sensitive to lower glucose levels because it lacks the LID regulatory domain and whose expression in the absorptive state is greater and maintained over time compared to ChREBP isoform [142]. Therefore, the actions of the isoforms α and β of ChREBP on the liver are not yet known and meanwhile, neither activation nor repression of ChREBP in this organ have yet demonstrated to be viable pharmacological strategies.

One of the mechanisms by which ChREBP promotes insulin sensitization in the liver could be through the expression of stearoyl-CoA desaturase-1 gene (Scd-1), which transforms saturated fatty acids into monounsaturated ones (MUFA), avoiding the repressive effect of saturated fatty acids on Akt phosphorylation in insulin signaling. This has been verified by lipidomic studies showing an increase in unsaturated fatty acids in hepatic steatosis, a condition where there is also an increase in the expression of ChREBP [135]. Another possible mechanism is the expression of the hepatokin FGF21 mediated by ChREBP. This factor, which is increased in obesity, improves glucose tolerance and reduces hypertriglyceridemia [143]. Its expression was initially described as a response to fasting through the activation of PPARα [144]. Later, it was found that carbohydrates also increase the expression of FGF21 through ChREBP, as an adaptation to increased caloric intake [145]. Today, FGF21 is known to be a key signal in tissue coordination and response to different nutritional stress such as fasting, ketogenic diets, amino acid deprivation and carbohydrate intake [146]. One of the recently described effects of FGF21 is its action on the central nervous system, reducing the preference for sugar and alcohol. This may be another intelligent adaptive response to the state of obesity. However, since the mechanism in mice is based on the reduction of dopamine levels, the possibility arises that dopamine analogues under clinical trials may have adverse effects on intake preference or other rewarding stimulus-driven behaviors [147]. Along with its action on macronutrient preference, FGF21 appears essential in the metabolic response to fructose mediated by ChREBP. In fact, the absence of FGF21 leads to liver disease when an organism is challenged with high fructose diets [148].

Regarding the role of ChREBP in fructose metabolism, research focused on this topic raised in parallel with the suggested key role of this factor in non-alcoholic fatty liver induced by high fructose intake. The importance of ChREBP in fructose metabolism was evidenced in a key experiment with ChREBP knocked-out mice fed a high fructose diet, in which the animals were unable to incorporate fructose and died after a few days [134]. The molecular mechanism underlying this fructose intolerance is currently known. ChREBP induces the expression of glucose transporters such as GLUT5 and GLUT2 in enterocytes, and enzymes such as ketohexokinase (KHK) in the liver, which are essential for the incorporation and metabolism of fructose, respectively [149]. On the other hand, ChREBP also seems to contribute to the negative effects of fructose, such as hyperglycemia and hepatic steatosis. Once fructose is absorbed in the intestine, it is rapidly transformed into glucose, avoiding the formation of advanced glycation products (AGEs) and therefore reducing this nocive effect [150]. Then, de novo lipogenesis in liver could be stimulated by ChREBP-mediated activation of lipogenic enzymes and by the presence of the required intermediates [151]. Furthermore, fructose causes increased expression of the glucose-6-phosphatase (G6pc) [152] and the glucagon receptor [153] genes through ChREBP favoring gluconeogenesis and glycogenolysis, respectively. Interestingly, both pathways trigger negative feedback on ChREBP by decreasing the levels of G6P by the action of G6pc and increasing the levels of the PKA repressor from glucagon signaling. In short, ChREBP promotes intestinal absorption of fructose, which is lower than glucose absorption [154], preventing the complications of its accumulation in the colon and the subsequent metabolic utilization by colonic microbiota, which could lead, for example, to inflammatory bowel disease [155]. Likewise, ChREBP favors the use of fructose in the liver and, although it increases the production of glucose and fatty acids, it establishes regulatory mechanisms to reduce the intake of sugars or the activation of ChREBP itself. Despite the contribution of ChREBP in fructose-induced fatty liver, this factor has a very important role in preserving liver function in fructose-high diets possibly by decreasing endoplasmic reticulum stress and repressing overactive cholesterol synthesis resulting in less hepatocytes apoptosis [156].

In adipose tissue, ChREBP is also highly expressed and promotes lipogenesis de novo. However, the adipose tissue-specific ChREBP knocked-out mouse is associated with widespread insulin resistance, even greater if mice are fed with a fructose rich diet. This relationship is explained by the inhibition of GLUT4 translocation to the membrane in adipocytes and the decrease in the levels of palmitic acid esters of hydroxy stearic acids (PAHSAs) in these knocked-out mice [157]. PAHSAs are fatty acid derivatives that promote insulin secretion, insulin-dependent glucose uptake in adipocytes and reduce inflammation, making them a promising therapy for T2DM [158]. Longitudinal studies confirm this relationship, where obese people show less expression of ChREBP-β in white adipose tissue, less de novo lipogenesis and greater tendency to insulin resistance [159]. With regard to brown adipose tissue, ChREBP-β is activated by cold to promote lipogenesis in order to allow thermogenesis [160]. Consequently, ChREBP participates in the main functions of white and brown adipose tissue.

In pancreas, glucose promotes the proliferation of pancreatic β cells through ChREBP, inducing the expression of cyclins [161]. However, in a situation of chronic hyperglycemia, the activation of ChREBP-β induces lipogenesis through the activation of FAS and redox imbalance by the thiorredoxin-interacting protein (TXNIP), leading to increased oxidative stress, reduced transcription and secretion of insulin and apoptosis of pancreatic β cells, evidencing the typical response of glycolipotoxicity [162].

Other actions of ChREBP have been described in the regulation of the circadian cycle by increasing the expression of internal clock regulators such as Kruppel-like factor 10 (KLF10) or helix-loop-binding protein 2 (BHLHB2), which in turn inhibit the transcriptional activity of ChREBP [163]. This is especially relevant given the association of altered circadian rhythms with metabolic diseases.

Based on the above-mentioned evidence, it can be said that a state of overfeeding causes a deregulation in glucose metabolism among the main tissues, partly driven by ChREBP [136]. In pancreas and liver, overexpression of ChREBP-β leads to overactivation of glucose input and lipid synthesis, resulting in hepatic steatosis and apoptosis of pancreatic β cells. In contrast, in muscle (by overexpression of MondoA) and adipose tissue (by decreased expression of the ChREBP-β isoform) glucose uptake is reduced, thus limiting the contribution of these tissues to insulin sensitization. Undoubtedly, ChREBP has a central and pleiotropic role in metabolic regulation, which, in turn, means that its study as a therapeutic target in diseases such as T2DM or fatty liver may involve adverse effects. Even so, further study of ChREBP could help to clarify all its functions and discover how the pharmacological modulation of this factor, or its effectors on signaling, could provide health benefits.

## 4. Conclusions and Outlook

Lipids and carbohydrates (along with proteins, not covered in this review) are essential macronutrients in the diet that direct metabolic regulation. Of particular interest is how part of this metabolic response can be directly modulated by the macronutrients through macronutrient sensing molecules that act as transcription factors.

In the case of lipids there is some controversy about the involvement of some nuclear receptors while PPARs are preferred receptors for fatty acids, especially PUFAs. The three subtypes of PPAR carry out complementary and sometimes overlapping functions in the control of glucose and lipid metabolism in many tissues. This could be one of the reasons why drugs targeting these factors have adverse effects in some cases and undetermined in others and still do not represent the coveted magic bullet against obesity and T2DM. Perhaps combinatorial strategies or supplementation with unsaturated fatty acids, which are natural ligands of the three PPARs, could be safer and more effective drugs or nutraceuticals.

Regarding carbohydrates, ChREBP plays a truly pleiotropic role, partly due to the combination of isoforms and their MondoA counterpart, in the body’s energy regulation. The actions of these factors are crucial in the maintenance of homeostasis in normal situations, but they can mediate in the pathogenesis of some metabolic diseases in states of overfeeding. This means that a successful pharmacological intervention is still a long way off.

Both carbohydrates and lipids are good examples to study under a nutrigenomics approach, since these macronutrients (very relevant due to their high presence in the diet) induce the transcription of genes implicated in different metabolic pathways. Among these routes are the main ones of glucose and lipid metabolism, so these nutrients directly regulate their own metabolism achieving a fine and integrating control of these energy sources. An example of the remarkable interrelationship between ChREBP and PPAR in glucose and lipid metabolism points to the hepatokin FGF21, which is induced in different situations by both factors participating in metabolic regulation and even in intake behavior.

In addition to the complex metabolic homeostasis, the pathological deregulation is even less known. This review discusses how both PPAR and ChREBP could be implicated in the prevention of T2DM, while ChREBP is involved in hepatic steatosis (in a context of overfeeding) but preserves liver function. In other words, these transcription factors carry out an adaptive response to nonphysiological situations such as overeating in order to maintain glucose levels, the main energetic source. Possibly the decisive role of ChREBP and PPAR in metabolic regulation points to them as ideal therapeutic targets, but their variety of functions in different tissues makes it difficult to “hit the mark”. This does not mean that this idea of pharmacologic intervention should be abandoned; on the contrary, more research is needed in this field to have a better understanding of the activation and function of these factors in the body and their successful application in diseases.

In the coming years, a broad development of nutraceuticals can be foreseen due to the current great interest in their research. One possible group of new nutraceuticals discussed in this paper could be the PAHSAs, whose insulin-sensitizing action could be useful in the prevention of T2DM. Finally, the attractive line of nutrigenomics will be expanded as new molecular connections are discovered in the complex and not totally unraveled path from food to genes, and although it may encounter some stones in the road may possibly result in many benefits regarding the knowledge and treatment of diseases in the XXI century.

## Data Availability

Not applicable.
